# Bioinformatics-based analysis of SUMOylation-related genes in hepatocellular carcinoma reveals a role of upregulated SAE1 in promoting cell proliferation

**DOI:** 10.1515/med-2022-0510

**Published:** 2022-07-06

**Authors:** Yang Liu, Xiang Wang, Xingzhi Zeng, Yinghua Wu, Xinrong Liu, Juan Tan, Xiaoyan Li

**Affiliations:** Department of Pathology, The Third Xiangya Hospital of Central South University, Changsha, Hunan, 410013, China; Xiangya School of Medicine, Central South University, Changsha, Hunan, China; Department of General Surgery, The Second Xiangya Hospital of Central South University, Changsha, Hunan, China; Department of Blood Transfusion, Shanxi Province People’s Hospital, No. 29 Shuangtasi Street, Yingze District, Taiyuan, Shanxi, 030012, China; Department of Pathology, The Third Xiangya Hospital of Central South University, No. 138 Tongzipo Road, Yuelu District, Changsha, Hunan, 410013, China

**Keywords:** hepatocellular carcinoma, IGF2BP3, prognostic model, SAE1, SUMOylation

## Abstract

The function of small ubiquitin-like modifier (SUMO)-related genes in hepatocellular carcinoma (HCC) remains unclear. This study aimed to analyze the expression profile and prognostic relevance of SUMO-related genes using publicly available data. A set of bioinformatics tools and experiments were integrated to explore the mechanism of the genes of interest. The least absolute shrinkage and selection operator Cox regression analysis was used to construct a prognostic model. SUMO-2 and SUMO-activating enzyme subunit 1 (SAE1) were upregulated in HCC. The enrichment analysis indicated that SUMO-2 and SAE1 might regulate the cell cycle. The downregulation of SAE1 inhibited the proliferation of HCC cells, whereas the upregulation of the gene promoted cell proliferation. IGF2BP3 contributed to the upregulation of SAE1 in an N6-methyladenosine (m6A)-dependent way. Eventually, an SAE1-related risk score (SRRS) was developed and validated in HCC. SRRS could serve as an independent prognostic factor and predict the efficiency of transarterial chemoembolization in patients with HCC.

## 1 Introduction

Uncontrolled cell proliferation is an important hallmark of cancer. Many anticancer therapies are designed to target the aberrantly activated cell cycle and DNA replication [1]. For example, nucleoside analogues, which disturb DNA replication, have been in clinical use for decades and have become the cornerstone of chemotherapies [2,3]. CDK4/6 inhibitors, in combination with endocrine therapy, lead to significantly longer overall survival (OS) compared with endocrine therapy alone in patients with advanced-stage breast cancer [4]. However, not all types of cancer respond well to these strategies. Early studies have shown that chemotherapies bring minimal benefit to patients with advanced-stage hepatocellular carcinoma (HCC) [5,6]. Recently, Hsieh et al. reported that ribociclib and abemaciclib, two CDK4/6 inhibitors, had minimal effects on HCC cell viability, and palbociclib, another CDK4/6 inhibitor, exerted a cytotoxic effect on HCC in a CDK4/6-independent way [7]. These pieces of evidence suggested that the regulatory mechanism of the cell cycle in HCC still remained unclear.

SUMOylation is a well-recognized post-translational modification (PTM) during which a small ubiquitin-like modifier (SUMO) protein is conjugated to lysine residues of target proteins. Accumulating evidence suggest that SUMOylation participates in many cellular activities, such as transcription regulation, DNA repair, signal transduction, protein degradation, and so forth [8]. Three enzymatic steps occur during SUMOylation: SUMO activation catalyzed by SUMO-activating enzyme E1, SUMO conjugation to conjugating enzyme E2 (Ubc9), and SUMO conjugation to substrate catalyzed by E2 and SUMO ligases E3 [9]. The modification changes the molecular surface of target proteins, which may affect protein–protein interactions, activity, stability, or cellular localization of substrates [10,11]. In mammalian cells, the SUMO family consists of four isoforms (SUMO1–4). SUMO-1 usually modifies substrates as a monomer, while SUMO-2/3 can form poly-SUMO chains [12]. SUMO-2 and SUMO-3 share 95% homology with each other, but they are only 45% identical to SUMO-1 [13]. SUMO-4 is usually nonconjugated under normal conditions [12].

Recent studies found that the dysregulation of SUMOylation contributed to the initiation and development of cancer [14,15,16,17], and that most SUMO-related genes, including SUMO-2 and SUMO-activating enzyme subunit 1 (SAE1), were overexpressed in many types of tumor [16,17,18]. For instance, SUMO-1-modified MAFB promoted colorectal cancer tumorigenesis via cell cycle regulation [15]. Yang et al. reported that SAE1 enhanced glioma growth by increasing SUMOylation and phosphorylation at Ser473 of Akt [16]. Du et al. found that the level of SUMO E1 was higher in colorectal cancer than in corresponding normal tissues. Further investigation revealed that SUMOylation enhanced the stability of Oct-1, a transcription activator of ALDH1A1 [17], which functioned in retinoic acid cell signaling [19]. Furthermore, the inhibition of SUMOylation impeded cell proliferation in various cancer cell lines [20], suggesting that SUMOylation might serve as a valuable therapeutic target.

Some studies demonstrated the importance of SUMOylation in the progression of HCC. For instance, SUMOylation of many proteins, such as liver kinase B1 (LKB1) [21], Shp2 (a protein tyrosine phosphatase) [22], and methyltransferase-like 3 (Mettl3) [23], has been reported to promote the growth of HCC. Besides, SUMO-related proteins may enhance the expression of several oncogenes. Guo et al. reported that SUMO-1 was overexpressed in HCC cell lines and promoted cancer cell proliferation by enhancing the expression of Bcl-2 and c-Myc [24]. However, the specific role of SUMO-related genes in HCC is not clear hitherto. In this study, we systematically investigated the role of SUMO-related genes in HCC and found that SUMO-2 and SAE1 were highly expressed in the disease and participated in regulating DNA replication and cell cycle. Since SAE1 initiates the process of SUMOylation, we further focused on the regulation and function of SAE1 in this disease. Immunohistochemistry staining (IHC) and *in vitro* experiments further confirmed the expression pattern and pro-proliferation function of SAE1 in HCC. Insulin-like growth factor 2 mRNA-binding protein 3 (IGF2BP3), an RNA N6-methyladenosine reader, exerted an essential impact on the upregulation of SAE1. Finally, an SAE1-related risk score (SRRS) was constructed and validated in HCC. The SRRS could serve as an independent prognostic factor and be used to predict the efficiency of patients with HCC receiving transarterial chemoembolization (TACE).

## 2 Materials and methods

### 2.1 Public data acquisition and processing

Normalized RNA-seq data (HTSeq-FPKM), phenotype information, and survival data of the LIHC, cervical squamous cell carcinoma and endocervical adenocarcinoma (CESC), lymphoid neoplasm DLBC, pancreatic adenocarcinoma (PAAD), skin cutaneous melanoma (SKCM), thymoma (THYM), glioblastoma multiforme (GBM), and brain lower-grade glioma projects of The Cancer Genome Atlas (TCGA) were downloaded from the GDC hub of UCSC xena website (http://xena.ucsc.edu/public) on August 14, 2020. Normalized gene expression data and donor information of the liver cancer project (code: LIRI_JP) of the International Cancer Genome Consortium (ICGC) were downloaded from the ICGC data portal (https://icgc.org/). Normalized gene expression data of the GSE64041, GSE14520/GPL3921, and GSE104580 datasets (series matrix file) were downloaded from the Gene Expression Omnibus database through the GEOquery package in the R software (version 3.6.2). All the data were processed as reported in previous studies [25,26]. Besides, ethical approval was not required for these data because they were deposited in public databases.

### 2.2 Human tissue and immunohistochemistry (IHC) staining

The tumor and paired tumor-adjacent normal tissues of patients with HCC were collected in the Third Xiangya Hospital and processed as a tissue microarray. The use of human tissues was approved by the institutional review board of the Third Xiangya Hospital, Central South University (No: 2020-S584). Written informed consent form was obtained from patients whose tissues were used in this study.

HE staining and IHC staining were conducted on 8-μm paraffin-embedded sections. IHC staining was performed using a Ventana BenchMark LT Automated IHC Stainer (Ventana Medical System, AZ, USA). The primary antibodies used in this study were rabbit polyclonal anti-IGF2BP3 (Proteintech, 14642-1-AP, 1:200), rabbit polyclonal anti-HNRNPC (Proteintech, 11760-1-AP, 1:200), rabbit polyclonal anti-SAE1 (Proteintech, 10229-1-AP, 1:200), and rabbit monoclonal anti-Ki67 (Ventana Medical System, 790–4286). The slides were incubated with primary antibodies, followed by the application of Ventana Ultraview HRP Universal Multimer (Ventana Medical System, 253–4290). Two independent pathologists assessed all the IHC samples. The extent of cell staining (0–10% positive cells for 0; 11–50% positive cells for 2; 51–80% positive cells for 3; and >80% positive cells for 4) and the staining intensity (no staining for 0; slight staining for 1; moderate staining for 2; and strong staining for 3) were scored separately and then added to reflect the expression [27].

### 2.3 Gene expression profiling interactive analysis 2 (GEPIA2), UALCAN, and cBioPortal

GEPIA2 (http://gepia2.cancer-pku.cn/) web server, which integrates TCGA and GTEx gene expression data based on a uniform pipeline, was used to compare the transcript level of the genes of interest between HCC tumors and normal samples from TCGA and the GTEx projects. The GEPIA2 database was also used to examine the pan-cancer expression of the genes of interest. The UALCAN database (http://ualcan.path.uab.edu/) was used to compare the DNA methylation level of SAE1 between tumor and normal samples. The data of DNA methylation level of SAE1 was downloaded from the cBioPortal for Cancer Genomics (http://www.cbioportal.org/) for correlation analysis. The last database was also used to evaluate the genomic alteration status of SAE1 in HCC.

### 2.4 Enrichment analysis

Gene ontology (GO) and Kyoto Encyclopedia of Genes and Genomes (KEGG) enrichment analyses were performed by the clusterProfiler package in R version 4.1.0. Gene Set Enrichment Analysis (GSEA) was performed by GSEA software (version 4.1.0). These pathways were considered to be significantly enriched when the following criteria were met: nominal *P*-value <0.05, false discovery rate *q*-value <0.25, and absolute normalized enrichment score >1.

### 2.5 Cell culture

HepG2 cells were obtained from the Chinese Academy of Sciences Cell Bank (Shanghai, China) and were confirmed to be free of mycoplasma before experiments. The cells were cultured in DMEM (ThermoFisher Scientific, USA) supplemented with 10% fetal bovine serum (FBS, ExCell) and incubated under humidified atmospheric conditions with 5% CO_2_ at 37°C. The expression vector of SAE1 and short hairpin RNA for SAE1 were purchased from Genechem Co., Ltd. For stable gene transfection, HepG2 cells were seeded overnight and then transfected with a SAE1-GV367-puro expression vector (OE), a sh-SAE1-GV493-puro expression vector (KD), or corresponding control vectors for the selection of puromycin-resistant cells.

### 2.6 Cell Counting Kit-8 (CCK-8) assay and cell cycle assay

For the CCK-8 assay, HepG2 cells were seeded in 96-well plates at a density of 2,000 cells per well and incubated for 1, 2, 3, 4, or 5 days. Then, 10 μL of CCK-8 (Dojindo Molecular Technologies, Japan) was added to each well, incubated for 4 h, and mixed gently on an orbital shaker for 2 min before the absorbance value (OD) of each well was measured at 450 nm. For the cell cycle assay, the cells were seeded on 6 cm diameter plates with DMEM containing 10% FBS and labeled using a cell cycle detection kit (Sigma, USA) following the manufacturer’s protocols. The DNA content of labeled cells was analyzed with FACS cytometry (Millipore, USA). The experiments were performed in triplicate.

### 2.7 Construction of SRRS

SAE1 and its related genes underwent the least absolute shrinkage and selection operator (LASSO) Cox regression analysis, which was achieved via the glmnet package in R. The LASSO Cox analysis generated four crucial genes, which further underwent multivariate Cox regression analysis to generate the corresponding coefficient. A new score was calculated by multiplying the normalized gene expression of each gene and its corresponding coefficient, and SRRS was calculated with the formula reported in a previous study, namely, SRRS = (score-Min)/absolute(Max) [28].

### 2.8 Statistical analysis

For *in vitro* experiments, data were presented as the mean value ± standard deviation. The Student *t* test was used to determine statistical significance between the two groups. Data were graphically displayed using GraphPad Prism v.8.0.1 for Windows (GraphPad Software, Inc., CA, USA). Correlation analysis, univariate and multivariate Cox regression, prognosis analysis, and time-dependent ROC analyses were performed in R. For survival analysis, the median expression value was used as the cutoff value in dividing patients into two groups. The following packages in R were used for data process and visualization: “tidyverse,” “survival,” “survminer,” “dplyr,” “plyr,” “survivalROC,” “rms,” “limma,” “timeROC,” “ggplot2,” and “ggpubr.” A *P* value less than 0.05 indicated a statistically significant difference (^*^, *P* < 0.05; ^**^, *P* < 0.01; ^***^, *P* < 0.001; ****, *P* < 0.0001).

## 3 Results

### 3.1 SUMO2 and SAE1 were upregulated in HCC

**Figure 1:**
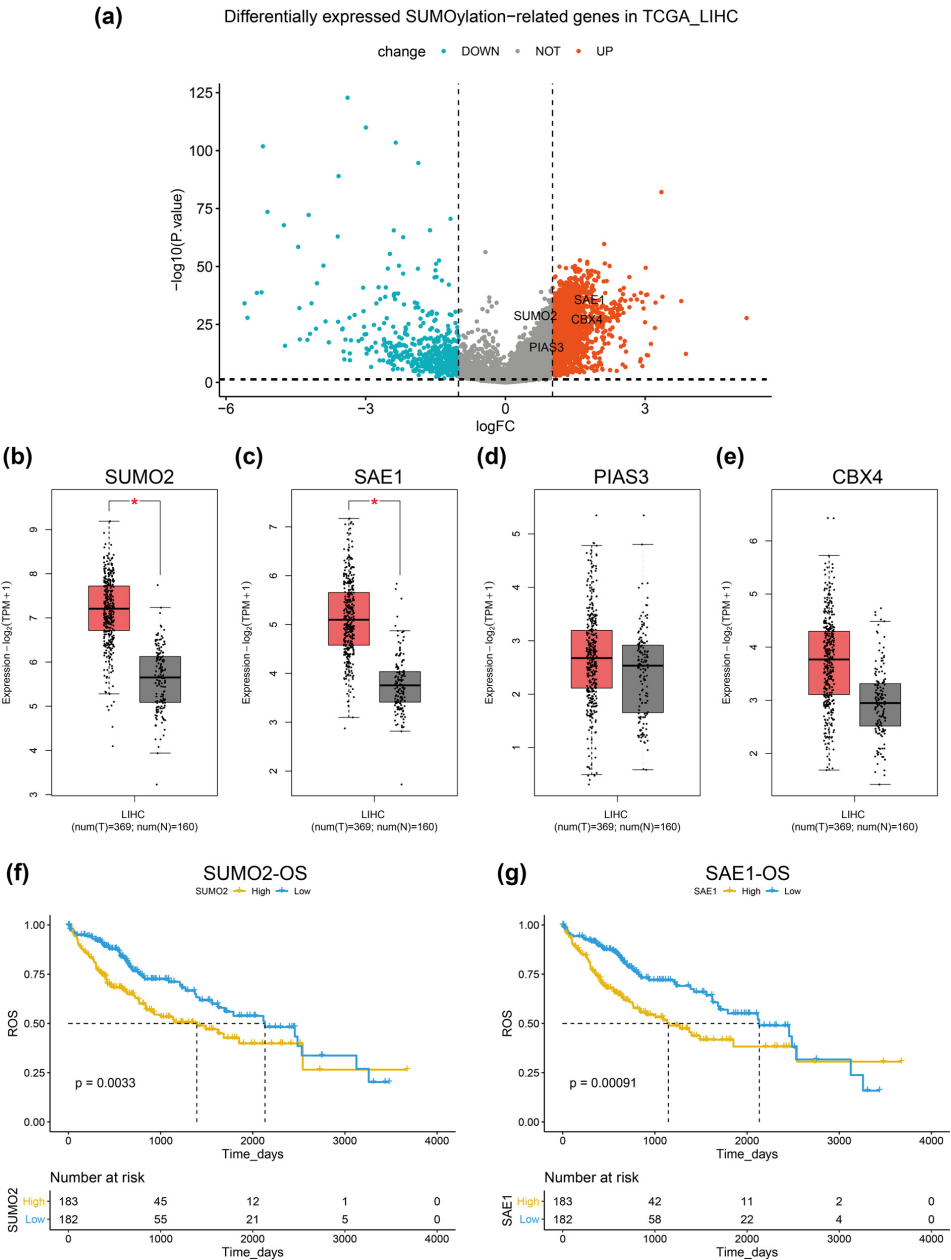
Expression profile of the SUMOylation-related genes in HCC. (a) Four differentially expressed SUMOylation-related genes were identified in HCC samples of the TCGA_LIHC dataset. (b–e) Comparison of SUMO2 (b), SAE1 (c), PIAS3 (d), and CBX4 (e) between HCC and normal livers in the GEPIA2 database. (f and g) Prognostic analysis of SUMO2 (f) and SAE1 (g) in patients with HCC from the TCGA_LIHC dataset.

**Figure A1:**
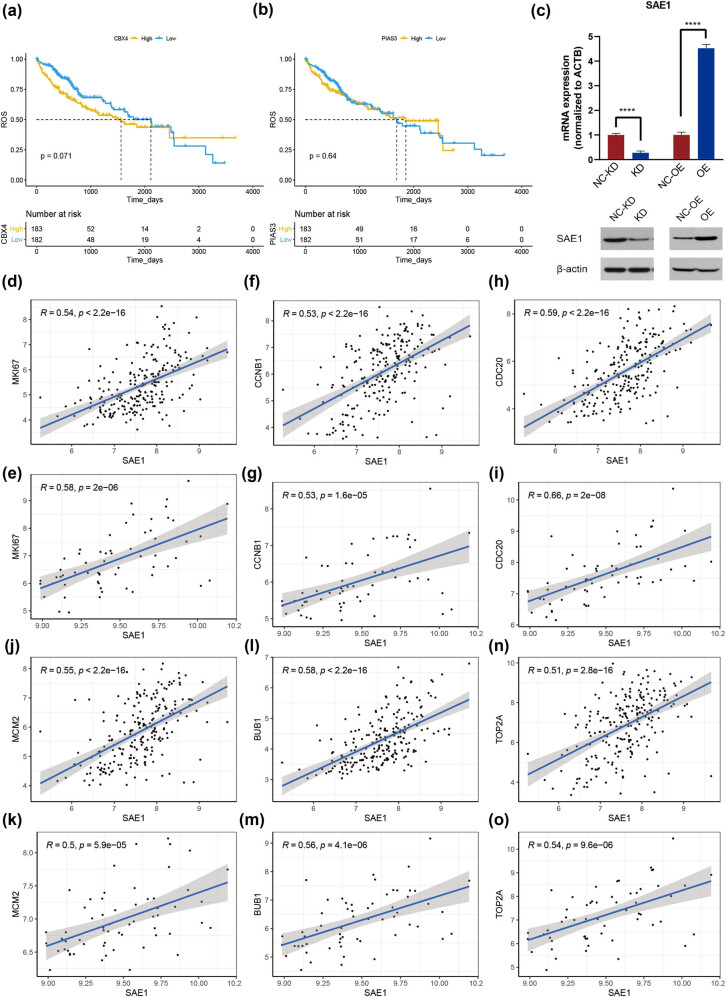
(a and b) Prognostic analysis of CBX4 (a) and PIAS3 (b) in HCC patients from the TCGA_LIHC dataset. (c) SAE1 was effectively silenced or over-expressed in HepG2 cells. (d–e) Correlation analysis between the expression of SAE1 and that of Ki67 in the GSE14520 (d) and GSE64041 (e) datasets. (f–g) Correlation analysis between the expression of SAE1 and that of CCNB1 in the GSE14520 (f) and GSE64041 (g) datasets. (h–i) Correlation analysis between the expression of SAE1 and that of CDC20 in the GSE14520 (h) and GSE64041 (i) datasets. (j–k) Correlation analysis between the expression of SAE1 and that of MCM2 in the GSE14520 (j) and GSE64041 (k) datasets. (l–m) Correlation analysis between the expression of SAE1 and that of BUB1 in the GSE14520 (l) and GSE64041 (m) datasets. (n and o) Correlation analysis between the expression of SAE1 and that of TOP2A in the GSE14520 (n) and GSE64041 (o) datasets.

We first identified all differentially expressed genes (DEGs) between tumor and tumor-adjacent normal samples in TCGA_LIHC dataset to understand the expression profile of SUMOylation-related genes in HCC. As shown in Figure 1a, four SUMOylation-related genes, namely, SUMO2, PIAS3, SAE1, and CBX4, were identified to be upregulated DEGs in the dataset. Next we compared the expression of the aforementioned four genes between HCC and normal livers from the TCGA and GTEx databases in the GEPIA2 web server. Using the default cutoff value of the GEPIA2 (|Log2FC| cutoff = 1, and *q*-value cutoff = 0.01), we found that SUMO2 and SAE1 were significantly upregulated in the tumor samples compared with normal livers. At the same time, PIAS3 and CBX4 showed no difference in expression between the two groups (Figure 1b–e). Further, we found that patients with HCC having the high expression of SUMO2 and SAE1 showed significantly shorter OS time (Figure 1f–g), while no prognostic significance was observed for PIAS3 and CBX4 expression in the disease (Figure A1a and b).

**Figure 2:**
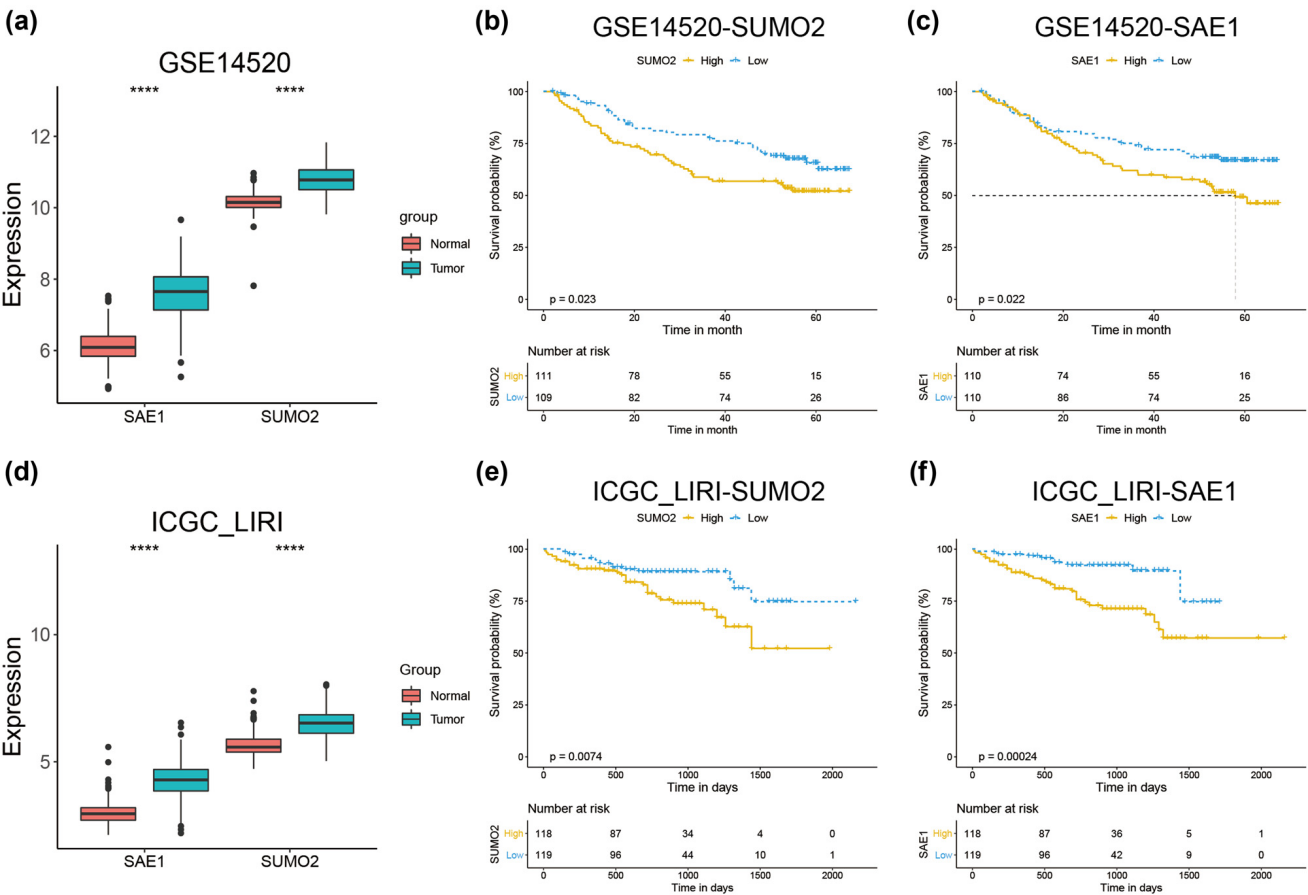
Validation of the expression pattern and prognostic significance of SUMO2 and SAE1. (a) Expression of SUMO2 and SAE1 in tumor and normal samples of the GSE14520 dataset. (b and c) Prognostic analysis of SUMO2 (b) and SAE1 (c) in patients with HCC from the GSE14520 dataset. (d) Expression of SUMO2 and SAE1 in tumor and normal samples of the ICGC_LIRI dataset. (e and f) Prognostic analysis of SUMO2 (e) and SAE1 (f) in patients with HCC from the ICGC_LIRI dataset. *P* < 0.05 was statistically significant, ns: not significant, ^*^ *P* < 0.05, ^**^ *P* < 0.01, ^***^ *P* < 0.001, ^****^ *P* < 0.0001.

Further, we validated the expression profile and prognostic relevance of SUMO2 and SAE1 in the GSE14520 and the ICGC_LIRI cohorts. In these two independent cohorts, both SUMO2 and SAE1 showed a significantly higher expression in tumor samples than in normal ones (Figure 2a and d). Also, the high expression of these two genes was associated with a worse prognosis in the disease (Figure 2b–f).

### 3.2 SUMO2 and SAE1 might regulate the cell cycle in HCC

**Figure 3:**
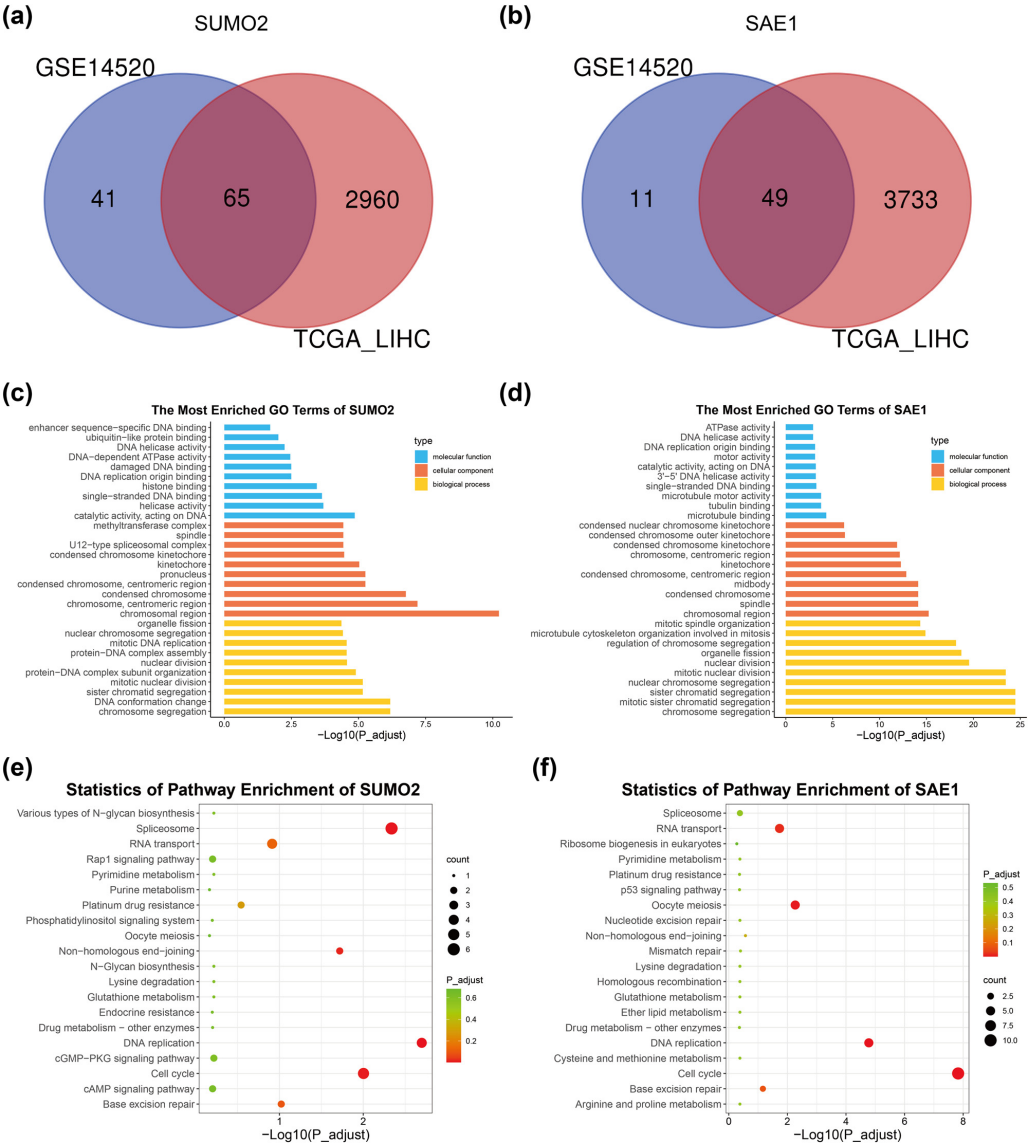
Enrichment analysis of SUMO2- and SAE1-related genes. (a and b) Identification of SUMO2-related (a) and SAE1-related (b) genes. (c and d) GO analysis of SUMO2-related (c) and SAE1-related (d) genes. (e and f) KEGG analysis of SUMO2-related (e) and SAE1-related (f) genes.

We conducted a correlation analysis between the expression of SUMO2 or SAE1 and that of other genes in tumor samples of the TCGA_LIHC and GSE14520 datasets to understand the function of SUMO2 and SAE1 in HCC. Genes highly associated with SUMO2 or SAE1 (Spearman correlation coefficient >0.5 and *P* < 0.05) were regarded as SUMO2-related or SAE1-related genes, respectively. As shown in Figure 3a and b, 65 SUMO2-related and 49 SAE1-related genes were shared in the TCGA_LIHC and GSE14520 datasets. The enrichment analysis showed that SUMO2-related genes were significantly enriched in the “enhancer sequence-specific DNA binding,” “ubiquitin-like protein binding,” “DNA-dependent ATPase activity,” “damaged DNA binding,” “single-stranded DNA binding,” “mototic nuclear division,” and “mitotic DNA replication” terms based on GO analysis (Figure 3c), and in the “DNA replication,” “spliceosome,” “cell cycle,” and “nonhomologous end-joining” according to the KEGG analysis (Figure 3e). Besides, SAE1-related genes were significantly enriched in the “chromosome segregation,” “mitotic sister chromatid segregation,” “mitotic nuclear division,” “organelle fission,” “microtubule cytoskeleton organization involved in mitosis,” “mitotic spindle organization,” “single-stranded DNA binding,” and “3′-5′ DNA helicase activity” according to GO analysis (Figure 3d), and in “cell cycle,” “DNA replication,” “oocyte meiosis,” and “RNA transport” pathways based on KEGG analysis (Figure 3f). Taken together, SUMO2 and SAE1 might play essential roles in regulating the cell cycle and DNA replication.

### 3.3 Pan-cancer analysis of SAE1

**Figure 4:**
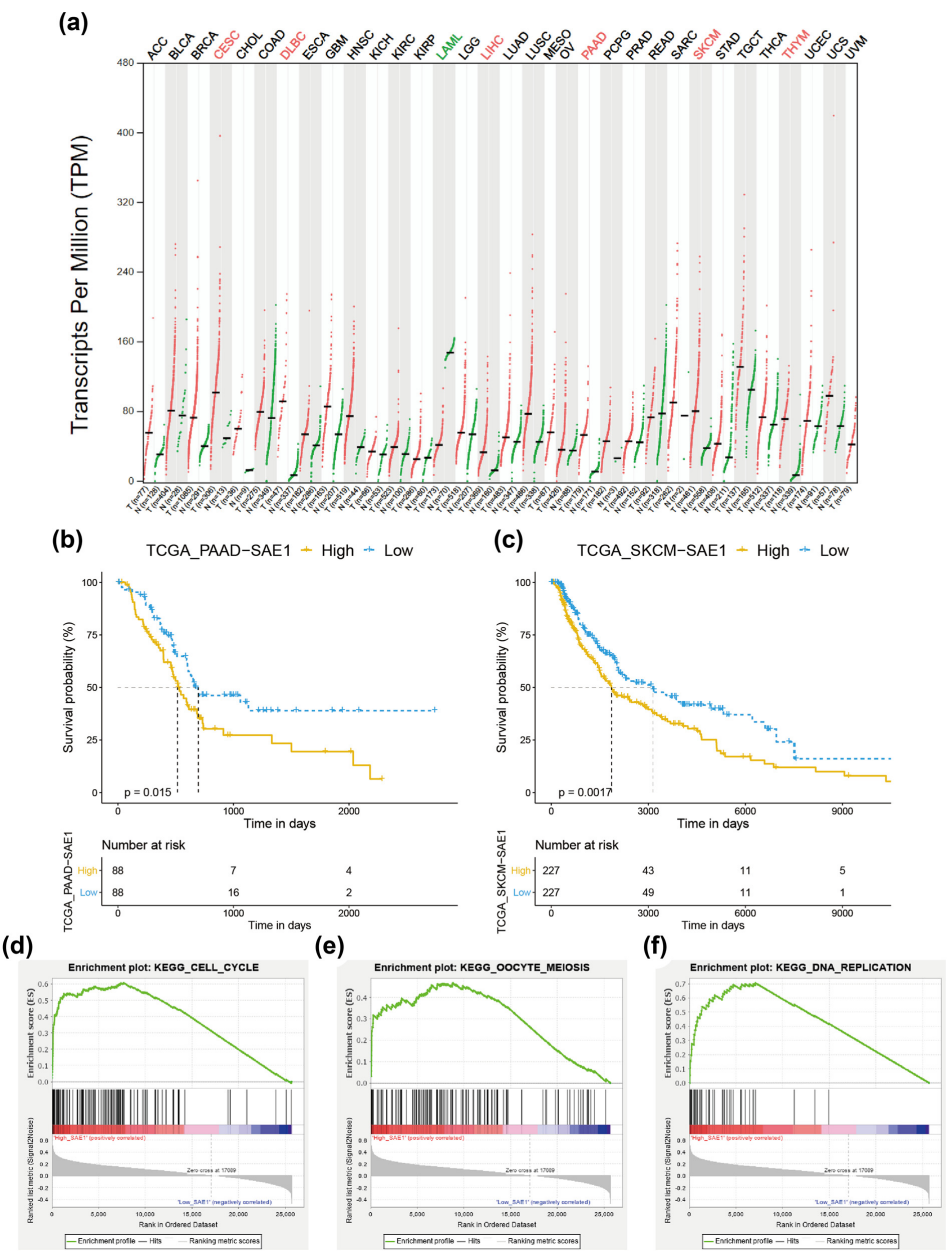
Pan-cancer analysis of SAE1. (a) Expression pattern of SAE1 across 33 types of tumor. (b and c) Prognostic analysis of SAE1 in cancer patients from the TCGA_PAAD (b) and TCGA_SKCM (c) datasets. (d–e) GSEA of low-SAE1 and high-SAE1 subgroups of the TCGA_PAAD and TCGA_SKCM datasets. Note: ACC (adrenocortical carcinoma), BLCA (bladder urothelial carcinoma), BRCA (breast invasive carcinoma), CESC (cervical squamous cell carcinoma and endocervical adenocarcinoma), CHOL (cholangiocarcinoma), COAD (colon adenocarcinoma), DLBC (lymphoid neoplasm diffuse large B-cell lymphoma), ESCA (esophageal carcinoma), GBM (glioblastoma multiforme), HNSC (head and neck squamous cell carcinoma), KICH (kidney chromophobe), KIRC (kidney renal clear cell carcinoma), KIRP (kidney renal papillary cell carcinoma), LAML (acute myeloid leukemia), LGG (brain lower grade glioma), LIHC (liver hepatocellular carcinoma), LUAD (lung adenocarcinoma), LUSC (lung squamous cell carcinoma), MESO (mesothelioma), OV (ovarian serous cystadenocarcinoma), PAAD (pancreatic adenocarcinoma), PCPG (pheochromocytoma and Paraganglioma), PRAD (prostate adenocarcinoma), READ (rectum adenocarcinoma), SARC (sarcoma), SKCM (skin cutaneous melanoma), STAD (stomach adenocarcinoma), TGCT (testicular germ cell tumors), THCA (thyroid carcinoma), THYM (thymoma), UCEC (uterine corpus endometrial carcinoma), UCS (uterine carcinosarcoma), UVM (uveal melanoma). (d) (ES: 0.607, NES: 1.816, NOM *p*-val: 0.002); (e) (ES: 0.467, NES: 1.725, NOM *p*-val: 0.004); (f) (ES: 0.706, NES: 1.776, NOM *p*-val: 0.016).

A recent study showed that the overexpression of SUMO2/3 promoted the proliferation of HCC cells via upregulating the protein level of HSP27, and silencing SUMO2/3 in the cells impaired their proliferative activity [29], supporting the aforementioned enrichment results that SUMO2 might regulate the cell cycle in the disease. However, the relationship between SAE1 and cell cycle regulation was seldom investigated in tumors including HCC. We conducted a pan-cancer analysis of SAE1 by manipulating data from the TCGA database to understand whether the function of SAE1 in cell cycle regulation was confined to HCC or a general phenomenon in solid tumors. As shown in Figure 4a, SAE1 was significantly upregulated in CESC, lymphoid neoplasm diffuse large B-cell lymphoma (DLBC), liver hepatocellular carcinoma (LIHC), pancreatic adenocarcinoma (PAAD), SKCM, and THYM, based on the default cutoff value of the GEPIA2 (|Log2FC| cutoff = 1, and *q*-value cutoff = 0.01). Further, Kaplan–Meier survival analysis indicated that patients with PAAD or SKCM having high expression of SAE1 had a significantly shorter OS time compared with corresponding patients with low SAE1 expression (Figure 4b and c). We divided patients into two groups based on the median expression of SAE1 in these two datasets and conducted GSEA, a widely used method to shed light on the biological function of a specific gene, to understand the role of SAE1 in these two types of cancer [30]. The results showed that genes in the high-SAE1 group were enriched in “cell cycle,” “oocyte meiosis,” and “DNA replication,” suggesting a general role of SAE1 in the cell cycle regulation in the two types of cancer (Figure 4d–f).

### 3.4 Tissue chip analysis of SAE1

**Figure 5:**
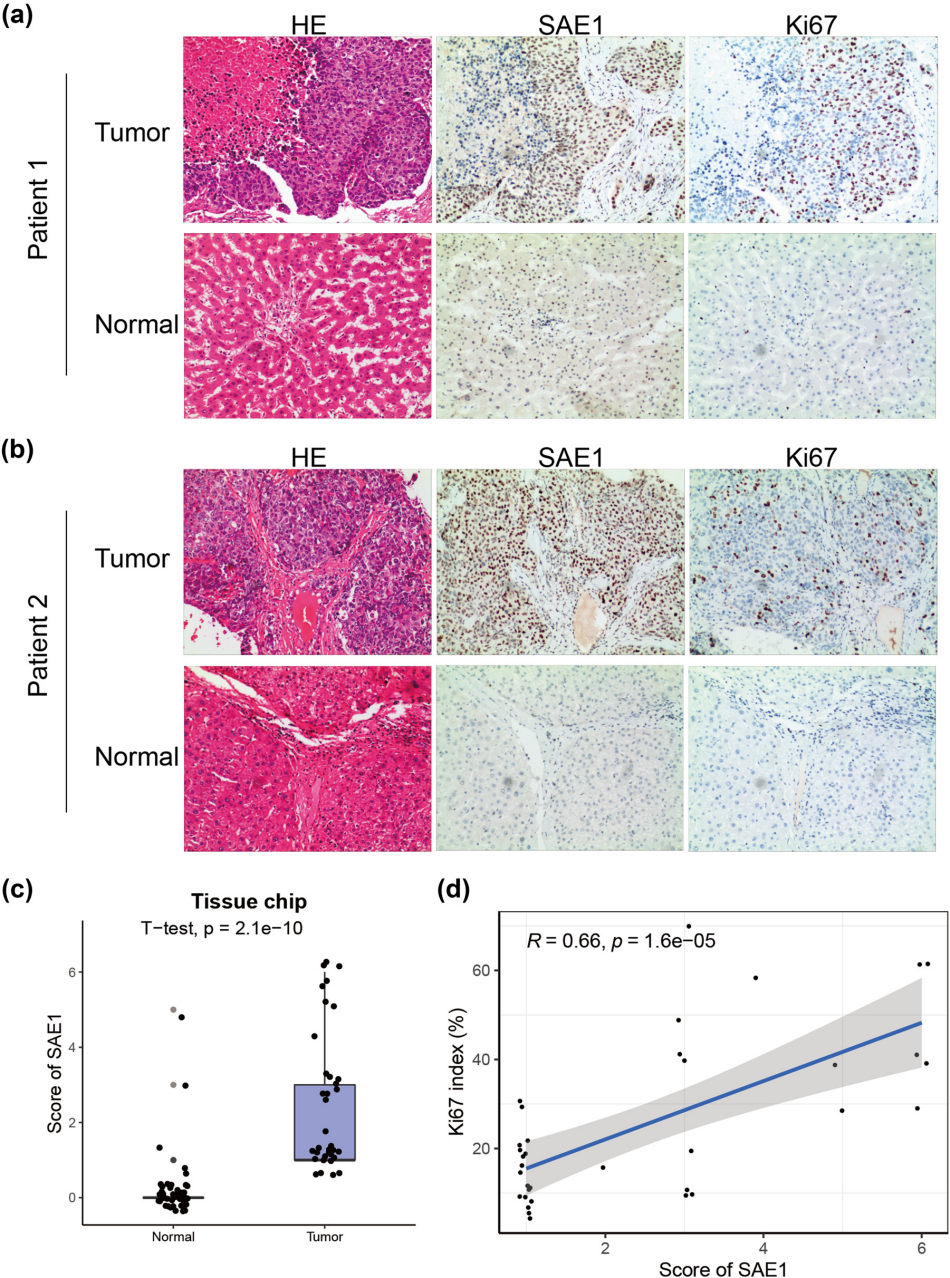
IHC staining of SAE1 on collected HCC samples (a and b) HE and IHC staining of SAE1 and Ki67 on collected HCC samples. (c) Comparison of scoring of SAE1 between HCC samples and tumor-adjacent normal livers. (d) Correlation analysis of the scoring of SAE1 and Ki67 index of the collected HCC samples.

We further evaluated the relationship between the expression of SAE1 and cell proliferation in HCC samples collected from our hospital. Further, 47 paired HCC samples and corresponding tumor-adjacent normal ones were subjected to the HE or IHC staining of SAE1 and Ki67. Two tumor samples were colon cancers with liver metastasis, 10 tumor samples exhibited no tumor cells based on HE staining, and 1 normal liver sample was destroyed during tissue slicing. Thus, these 13 samples were excluded from further analysis. Consistent with previous studies, SAE1 and Ki67 were predominantly located in the nucleus of cells (Figure 5a and b) [31,32], and SAE1 had significantly stronger staining in tumor samples than in normal livers (Figure 5c). In fact, most normal liver samples exhibited no staining of SAE1 (41/46), according to the scoring of two independent pathologists. In addition, the Ki67 index, a well-known marker for the evaluation of cell proliferation, had a strong positive correlation with the score of SAE1 (Figure 5d, *R* = 0.66, *P* = 1.6 × 10^−5^).

**Figure 6:**
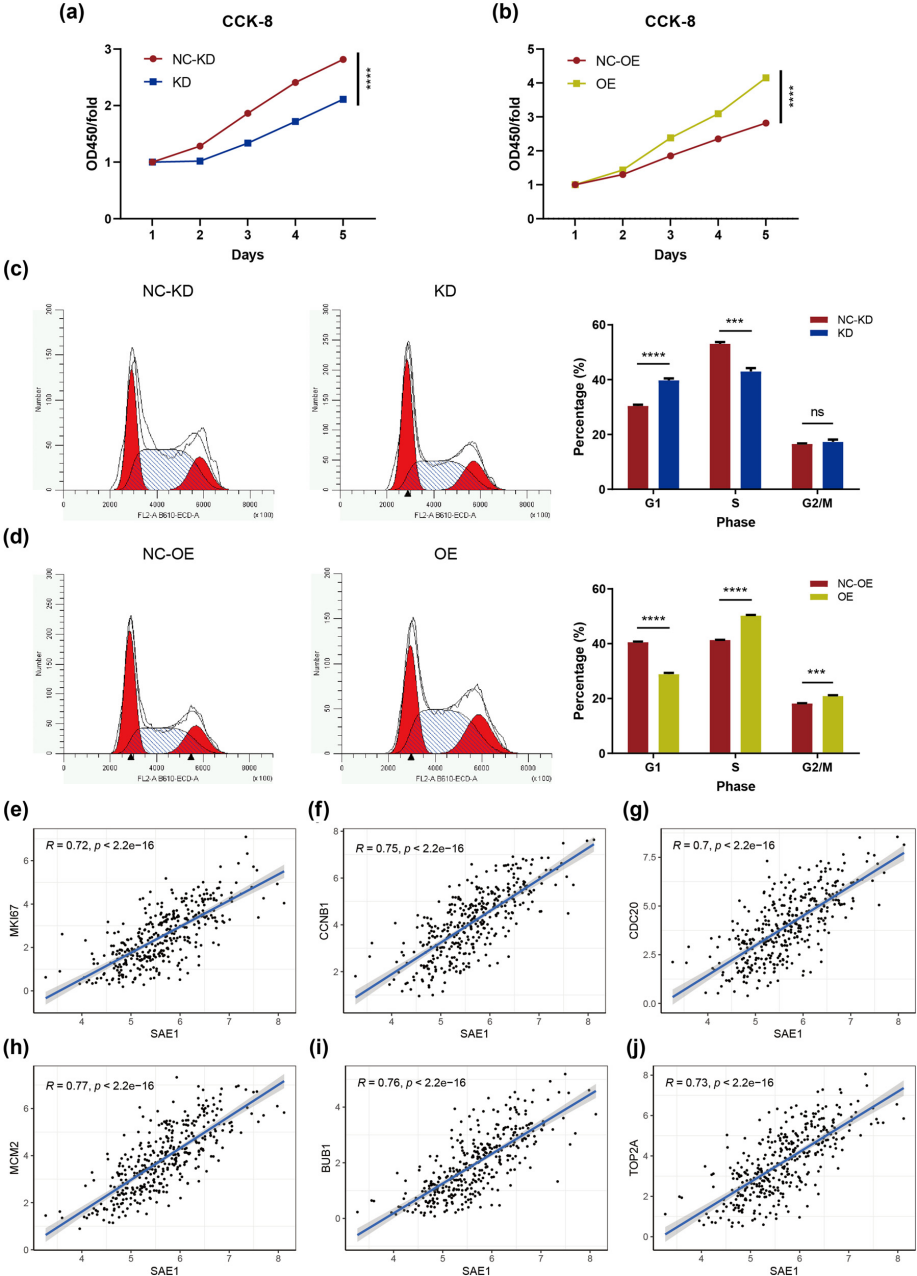
SAE1 promoted cell proliferation in HCC. (a and b) CCK-8 analysis of HepG2 cells with downregulated (a) or upregulated (b) expression of SAE1. (c and d) Cell cycle analysis of HepG2 cells with downregulated (c) or upregulated (d) expression of SAE1. (e–j) Correlation analysis between the expression of SAE1 and that of Ki67 (e), CCNB1 (f), CDC20 (g), MCM2 (h), BUB1 (i), or TOP2A (j) in the TCGA_LIHC dataset. *P* < 0.05 was statistically significant, ns: no significant, ^*^ *P* < 0.05^, **^ *P* < 0.01, ^***^ *P* < 0.001, ^****^ *P* < 0.0001.

We silenced or elevated the expression of the gene in HCC cells to confirm the function of SAE1 in regulating cell proliferation. As shown in Appendix 1c, the silencing strategy downregulated the expression of SAE1 to nearly 1/4 of that of the controlled cells, while the SAE1-GV367-puro expression vector led to more than 4 times expression of SAE1. As shown in Figure 6a and b, the downregulation of SAE1 impaired the proliferative capability of HCC cells, whereas the upregulation of the gene promoted cell proliferation. We further conducted a cell cycle assay and found that HCC cells with a decreased level of SAE1 had a higher percentage of cells in the G0/G1 phase and a lower percentage of cells in the S phase, compared with those in the control group (Figure 6c). In contrast, HCC cells with the elevated expression of SAE1 led to more cells in the S and G2/M phases and fewer cells in the G0/G1 phase (Figure 6d).

In addition, a strong positive correlation between SAE1 and Ki67 was also observed in three independent cohorts (Figure 6e, Figure A1d and e, *r* = 0.54–0.72). Besides, SAE1 also showed a strong positive correlation with a set of cell cycle regulators such as CCNB1 (Figure 6f, Figure A1f and g, *r* = 0.53–0.75), CDC20 (Figure 6f, Figure A1h–i, *r* = 0.59–0.70), MCM2 (Figure 6d, Figure A1j and k, *r* = 0.5–0.77), BUB1 (Figure 6b, Figure A1l and m, *r* = 0.56–0.76), and TOP2A (Figure 6e, Figure A1n and o, *r* = 0.51–0.73).

### 3.5 m6A modification contributed to the overexpression of SAE1 in HCC

**Figure 7:**
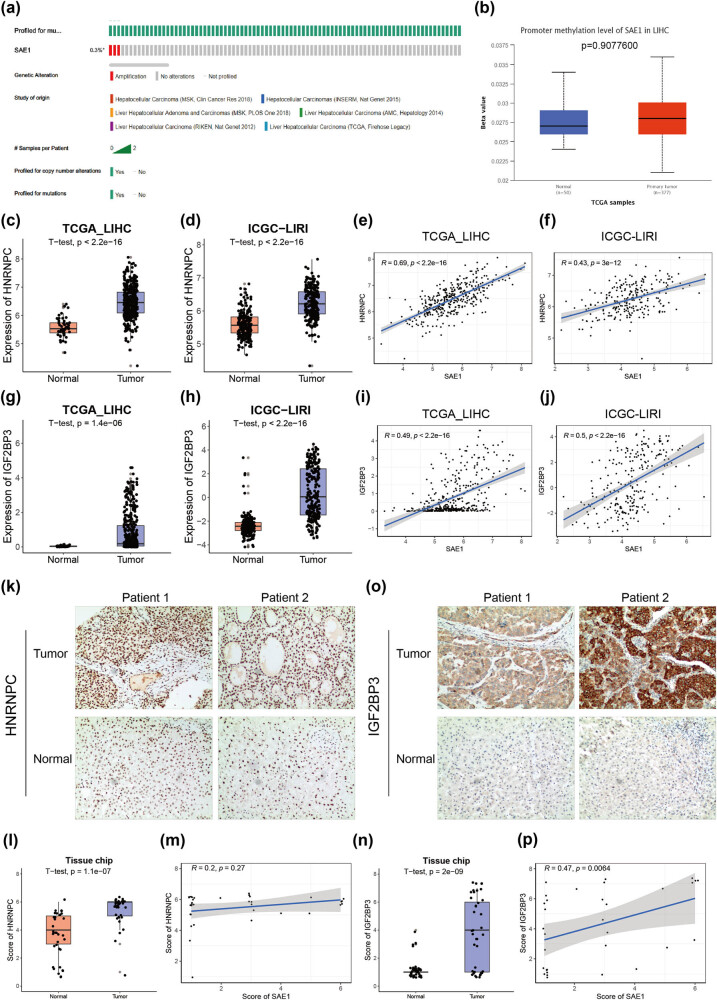
IGF2BP3 contributed to the upregulation of SAE1 in HCC. (a) Gene copy variation analysis of SAE1 in patients with HCC from six independent cohorts. (b) Promoter methylation level of SAE1 between tumor and normal livers. (c and d) Expression of HNRNPC between HCC samples and normal ones in the TCGA_LIHC (c) and ICGC_LIRI (d) datasets. (e and f) Correlation analysis between the expression of SAE1 and that of HNRNPC in the TCGA_LIHC (e) and ICGC_LIRI (f) datasets. (g and h) Expression of HNRNPC between HCC samples and normal ones in the TCGA_LIHC (g) and ICGC_LIRI (h) datasets. (i and j) Correlation analysis between the expression of SAE1 and that of HNRNPC in the TCGA_LIHC (i) and ICGC_LIRI (j) datasets. (k and l) IHC staining of HNRNPC on collected HCC samples. (m) Correlation analysis of the scoring of SAE1 and that of HNRNPC of the collected HCC samples. (n and o) IHC staining of IGF2BP3 on collected HCC samples. (p) Correlation analysis of the scoring of SAE1 and that of IGF2BP3 of the collected HCC samples.

**Figure A2:**
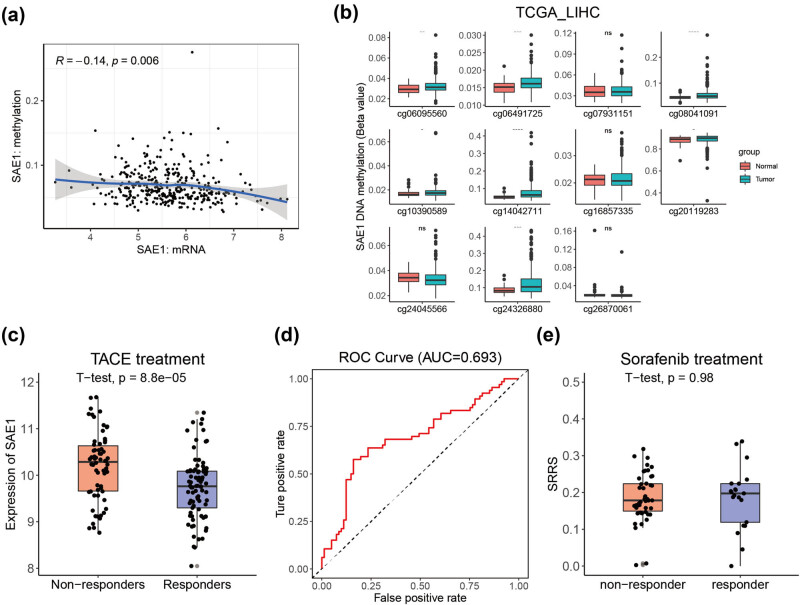
Construction and validation of SRRS. (a and b) LASSO Cox regression analysis of SAE1 and its related genes in the GSE14520 dataset, with the tuning parameter (*λ*) calculated based on partial likelihood deviance with tenfold cross-validation. (c and d) Kaplan–Meier plots (c) and time-dependent ROC analysis (D) of the SRRS regarding OS and survival status in the GSE14520 cohort. (e and f) Kaplan–Meier plots (e) and time-dependent ROC analysis (f) of the SRRS regarding OS and survival status in the TCGA_LIHC cohort. (g and h) Results of the univariate (g) and multivariate (h) Cox regression analyses regarding OS in the TCGA_LIHC cohort. (i) Comparison of SRRS between responders and nonresponders receiving TACE treatment. (j) ROC analysis of SRRS in predicting response to TACE treatment.

The aforementioned analysis indicated that SAE1 was overexpressed in HCC and promoted cell proliferation. We first evaluated the genetic alteration status of SAE1 in HCC to understand how the gene was upregulated in this disease. As shown in Figure 7a, only 0.3% of patients with HCC had the gene amplification of SAE1 and no alteration was observed in the remaining 99.7% of patients, according to the analysis across 6 HCC cohorts in the cBioPortal database. Next we examined whether the expression of SAE1 was affected by DNA methylation in its promoter regions. However, no difference was found in the promoter methylation level of SAE1 between tumor and normal livers (Figure 7b, *P* = 0.9077600), and only a weak negative correlation was observed between the DNA methylation level of SAE1 and the transcriptional level of the gene (Figure A2a, *r* = –0.14, *P* = 0.006). We analyzed the methylation level of all SAE1 CpG sites between normal and tumor samples in the TCGA_LIHC dataset to rule out the possibility that methylation at a specific CpG site of SAE1 might be strongly correlated with SAE1 mRNA expression. Considering that DNA methylation is mostly associated with transcriptional repression [33], we expected that hypomethylation occurred in the SAE1 CpG site in tumor samples because SAE1 was upregulated in HCC. However, except cg07931151, cg16857335, cg24045565, and cg26870061 (where no difference was observed in the methylation level between tumor and normal samples), significant hypermethylation was observed in the remaining seven CpG sites of SAE1 in tumors compared with normal tissues (Figure 2b), suggesting that DNA methylation was not likely to be the factor that caused the elevated expression of SAE1 in HCC.

Besides genetic alteration and DNA methylation, RNA modification is another essential mechanism in regulating mRNA expression and subsequent translation [34]. To date, more than 100 modifications have been found in RNA, and m6A is one of the most common and abundant modifications in eukaryotes [35,36]. We queried the M6A2Target (http://m6a2target.canceromics.org/), a comprehensive database for the target genes of writers, erasers, and readers (WERs) of m6A modification, to explore whether m6A modification played a role in the mRNA level of SAE1 [37]. Three m6A-related WERs are found to affect the expression of SAE1 in an m6A-dependent way, but only the effect of HNRNPC and IGF2BP3 reached significance, based on the adjusted *P* value of RNA-seq experiments (Table 1). Knocking down HNRNPC and IGF2BP3 in cancer cells led to decreased levels of SAE1, probably through the regulation of mRNA stability [38]. Indeed, we found that HNRNPC was significantly upregulated in HCC compared with normal tissues (Figure 7c and d). Besides, a significant positive correlation was observed between the expression of HNRNPC and that of SAE1 in tumor samples of both the TCGA_LIHC and ICGC_LIRI datasets (Figure 7e and f, *r* = 0.43–0.69). Similarly, a significantly higher expression of IGF2BP3 was also observed in HCC (Figure 7g and h), and a strong correlation was observed between the expression of IGF2BP3 and that of SAE1 (Figure 7i and j, *r* = 0.49–0.5). We further conducted IHC staining of IGF2BP3 and HNRNPC in our collected tissue samples. Both these genes had a considerably increased expression in HCC compared with adjacent normal livers (Figure 7k–p). The staining score of IGF2BP3 showed a significantly positive correlation with that of SAE1 (Figure 7q, *r* = 0.47, *P* = 0.0064); however, no significant correlation was observed between the staining score of SAE1 and that of HNRNPC (Figure 7m, *r* = 0.2, *P* = 0.27).

**Table 1:** WERs of m6A modification affecting the expression of SAE1 from the M6A2Target database

WER names	Cell line	Target gene	High-throughput method	Perturbation direction	Log2FC	Adj. *P* value	Perturbation effect	m6A dependence
METTL3	MDA-MB-231	SAE1	RNA-seq	Knock down	0.69	0.77	Upregulated	m6A dependent
HNRNPC	HEK293T	SAE1	RNA-seq	Knock down	–0.77	0	Downregulated	m6A dependent
IGF2BP3	HepG2	SAE1	RNA-seq	Knock down	–0.76	0	Downregulated	m6A dependent

### 3.6 Construction and validation of SRRS

**Figure 8:**
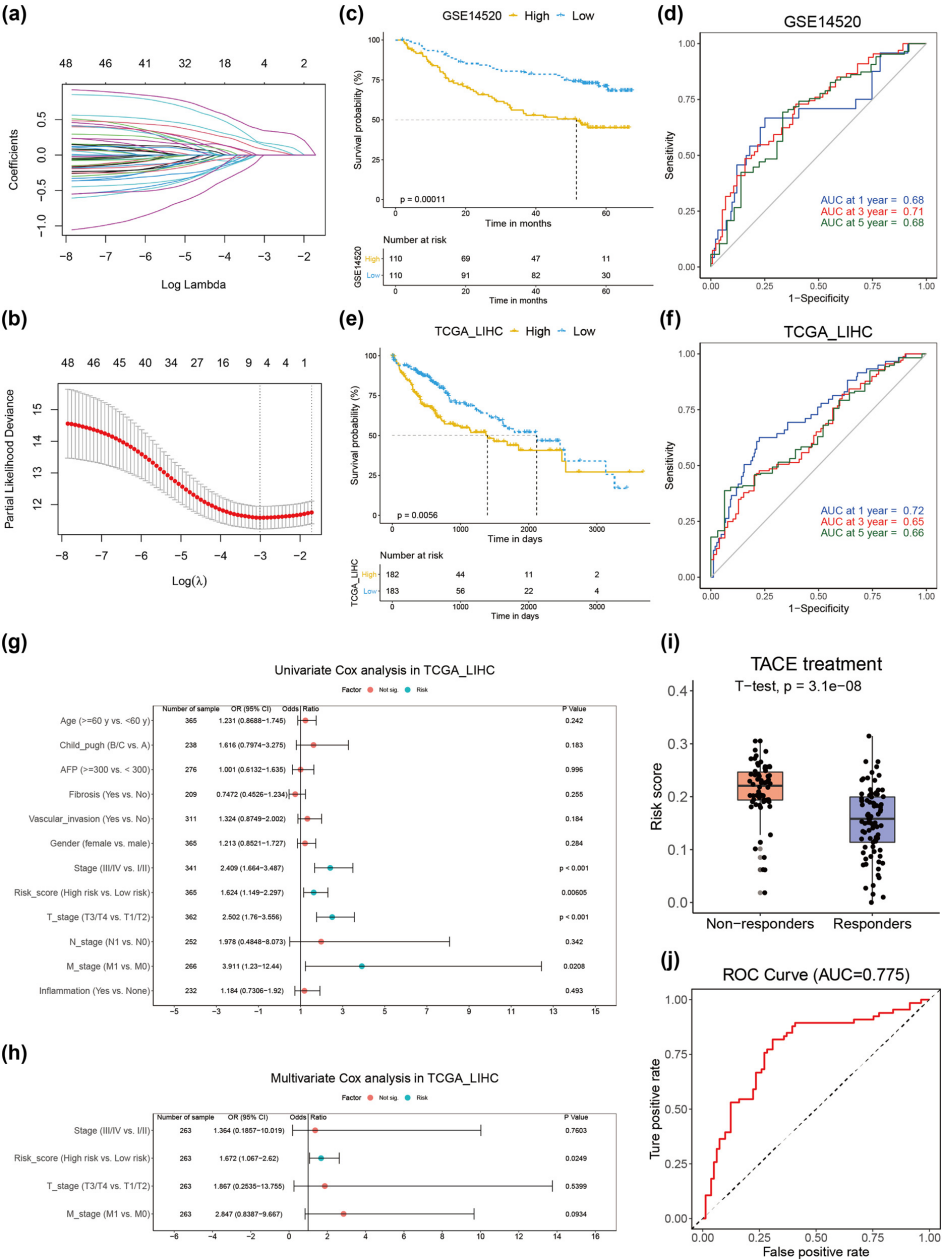
Construction and validation of SRRS. (a and b) LASSO Cox regression analysis of SAE1 and its related genes in the GSE14520 dataset, with the tuning parameter (*λ*) calculated based on partial likelihood deviance with tenfold cross-validation. (c and d) Kaplan–Meier plots (c) and time-dependent ROC analysis (D) of the SRRS regarding OS and survival status in the GSE14520 cohort. (e and f) Kaplan–Meier plots (e) and time-dependent ROC analysis (f) of the SRRS regarding OS and survival status in the TCGA_LIHC cohort. (g and h) Results of the univariate (g) and multivariate (h) Cox regression analyses regarding OS in the TCGA_LIHC cohort. (i) Comparison of SRRS between responders and nonresponders receiving TACE treatment. (j) ROC analysis of SRRS in predicting response to TACE treatment.

We first selected 4 crucial genes by inputting SAE1 and its 49 SAE1-related genes into a LASSO Cox regression model in the GSE14520 dataset to construct an SAE1-related prognostic model (Figure 8a and b). A novel score was calculated by multiplying the coefficient of each gene and its gene expression level, namely, score = 0.3860 × SNRPF + 0.2294 × CENPM + 0.1536 × MKI67 + 0.2538 × AMD1. The SRRS was calculated based on the aforementioned formula. The median value of the SRRS in each independent cohort was used as the cutoff value in dividing patients into the low- and high-risk populations. Prognostic analyses showed that patients with HCC in the low-risk subgroups had a significantly longer OS in the GSE14520 cohort (Figure 8c, *P* = 0.00011). Time-dependent receiver operator characteristic (ROC) analysis showed that the area under the curve (AUC) of SRRS reached 0.68 in 1 year, 0.71 in 3 years, and 0.68 in 5 years, suggesting a favorable predictive value (Figure 8d). The prognostic value of the SRRS was further validated in the TCGA cohort (Figure 8e and f). The univariate Cox analysis revealed that SRRS, T stage, and M stage had prognostic significance in HCC (Figure 8g). When these factors underwent multivariate Cox analysis, only SRRS was identified to be an independent prognostic factor (Figure 8h). We further evaluated whether SRRS could be used to predict response to anti-HCC treatment. TACE is an essential treatment for patients with early-stage and some advanced-stage HCC [39]. Patients with HCC who responded to TACE had significantly lower expression of SAE1 or lower SRRS (Figure 8i, Figure A2c). The AUC of SRRS in predicting the response to TACE reached 0.775 (Figure 8j), which was higher than that of SAE1 (AUC = 0.693), indicating that SRRS had better predictability. In addition, sorafenib is a well-established first-line therapy for patients with advanced-stage HCC [40]. However, SRRS was not different between responders and nonresponders in receiving sorafenib treatment (Figure A2e), suggesting that SRRS was not suitable in predicting the response of patients with HCC to this systematic treatment.

## 4 Discussion

HCC is the most common subtype of primary liver cancers [41], notoriously known for its poor prognosis. The 5-year survival rate of patients with liver cancer is only 18% [42], and the median life expectancy for patients with advanced-stage, unresectable HCC is only about 1 year [43]. Patients with unresectable HCC receiving lenvatinib, a newly approved first-line treatment, can only reach a median OS of 13.6 months [44]. Recently, cell cycle-targeting therapies have made progress in some cancers [4]. Nonetheless, these therapies failed in treating HCC [7]. How the cell cycle is regulated in HCC cells remains unclear. In this study, the role of SUMOylation-related genes was systematically investigated in HCC. SUMO-2 and SAE1 were highly expressed in tumor cells, and their low levels correlated with the longer survival of patients with HCC. GO and KEGG enrichment analyses revealed that the two genes were linked with both the cell cycle and DNA replication. Previously, the relationship between SUMO-2 and cell proliferation was reported by a study showing that SUMO2/3 promoted HCC cell proliferation and invasion through the modification and stabilization of HSP27 [29]. However, the role of SAE1 in cell cycle regulation remained unclear and became the focus of this study. The IHC staining on collected tumor samples from our hospital supported that SAE1 was overexpressed in HCC and strongly correlated with the Ki67 index. *In vitro* experiments demonstrated that HCC cells showed a tardy cell proliferation when SAE1 was silenced and promoted proliferation when the gene was overexpressed (Figure 6a and b). Knocking down the expression of SAE1 in HCC cells led to an increased percentage of cells in the G0/G1 phase, whereas upregulating the gene in HCC cells had a contrast function (Figure 6c and d). SAE1 showed a strong positive correlation with many cell cycle-related genes such as CCNB1, CDC20, MCM2, BUB1, and TOP2A (Figure 6 and Figure A1). A recent study reported that SAE1 is associated with dysregulated cancer metabolic signaling in HCC and contributed to the migration and invasion of HCC cells [45]. Although this study did not investigate the role of SAE1 in the cell proliferation of HCC, its *in vitro* experiments exhibited that the downregulation of SAE1 in HCC cells resulted in decreased protein levels of some cell cycle-related genes such as CDK4 and cyclin B1 [45], supporting the findings of this study that SAE1 regulated cell cycle and proliferation of HCC. In addition, pan-cancer analyses also demonstrated that SAE1 was related to the cell cycle in some other types of cancer (Figure 4), implicating that the role of SAE1 was probably not cancer type specific. Taken together, the overexpression of SAE1 in HCC contributed to aberrantly activated cell cycle and promoted tumor cell proliferation. Consequently, inhibiting SAE1 might be an effective strategy for anticancer treatment.

The mechanism of how SAE1 regulates cell cycle and promotes cell proliferation is yet unknown. Two possible mechanisms were proposed in this study. Since SAE1 is indispensable for the SUMOylation process, one possible mechanism could be that SAE1 affected the SUMO modification of proteins related to the cell cycle. In fact, the effect of SUMOylation on target proteins could be largely attributed to the interaction between SUMO proteins and SUMO-interaction motifs (SIMs). For instance, a study reported that SUMO2-modified proliferating cell nuclear antigen (PCNA), an important component of the DNA replication fork, could recruit histone chaperones CAF1- and FACT-containing SIMs, which then dislodged RNA polymerase II from the chromatin to resolve the transcription–replication conflict and promoted replication fork progression [46]. Besides, SUMOylation interacted with other PTMs, especially ubiquitylation, and participated in cell cycle regulation [9,47]. On the one hand, SUMOylation competed with or blocked ubiquitylation, enhancing the stability of target proteins. For example, the SUMOylation of CDK6 at Lys 216 blocked its ubiquitylation at Lys 147 and protected CDK6 from degradation [48]. SUMOylation of ZFHX3 increases its stability via preventing its ubiquitination, which is essential for ZFHX3 to promote cell proliferation in breast cancer cell lines [49]. On the other hand, SUMOylation promoted ubiquitylation. Eifler et al. reported that the SUMO2 modification of APC/C, a ubiquitin E3 ligase, increased its E3 ligase activity toward KIF18B, a kinesin that regulated chromosomal alignment and segregation. This might be explained by the SUMO–SIM interaction between SUMOylated APC/C and KIF18B [50]. Apart from affecting the SUMOylation process, SAE1 might also directly regulate the cell cycle via interaction with cyclin B1, BUB1, MCM2, and so forth. Recent studies found that SAE2 (UBA2) was overexpressed in many types of tumors and might function as a cell cycle regulator [51,52,53]. For instance, Jiang et al. found that the expression of UBA2 was significantly higher in non-small-cell lung cancer than in normal lung tissues, which was linked to tumor growth. Further research revealed that UBA2 might act as a cell cycle regulator by enhancing the expression of PARP1, MCM2, MCM3, and MCM7, which were all related to cell cycle and cell proliferation [51]. Since SAE1 and UBA2 were closely linked to each other, it was reasonable to hypothesize that SAE1 might also function as a cell cycle regulator via regulating the expression of genes related to the cell cycle. However, the exact mechanism of SAE1 in regulating the cell cycle and proliferation of tumor cells requires further exploration.

To understand how SAE1 is upregulated in HCC, we investigated copy number variation, DNA methylation, and RNA modification of the gene. By integrating 1,085 patients with HCC across 6 cohorts, we found that few patients (0.3%) had copy number amplification. Moreover, DNA methylation is a well-known form of DNA modification that usually inhibits gene expression [54]. However, the promoter methylation levels of SAE1 in HCC samples did not significantly differ from that in normal tissues, and only a weak correlation existed between the promoter methylation level of SAE1 and its mRNA expression. The methylation analysis of 11 CpG sites of SAE1 further indicated that several sites even exhibited a higher level of methylation in tumors. Taken together, these results inferred that neither copy number variation nor DNA methylation had a significant contribution to the upregulation of SAE1 in HCC. N6-methyladenosine (m6A) is the most abundant form of RNA modification in mammalian cells, which is involved in translation [55], mRNA splicing [56], mRNA degradation [57], and so forth. Based on the M6A2Target, a comprehensive database for the targets of m6A WERs [37], HNRNPC, and IGF2BP3 was proved to exert an impact on the expression of SAE1. Both these RNA-binding proteins were highly expressed in HCC samples, according to the analyses of RNA-seq data of publicly available datasets (TCGA_LIHC and ICGC_LIRI) and IHC staining on collected HCC samples (Figure 7). HNRNPC and IGF2BP3 preferentially bound to m6A-modified mRNAs and stabilized target mRNAs, thus promoting gene expression [38,58,59]. Besides, high expression of these two genes correlated with poor prognosis of patients with HCC [60,61]. However, the correlation analysis suggested that only the expression of IGF2BP3 had a significantly positive correlation with that of SAE1 (Figure 7m and q). Indeed, the knockdown experiments were conducted on an HCC cell line for IGF2BP3 and on the HEK293T cell line for HNRNPC (Table 1). Consequently, IGF2BP3 contributed to the upregulation of SAE1 in HCC, probably via the mRNA stabilization of the latter.

It should be pointed out that some other factors might also affect the expression of SAE1 in HCC. For example, Myc protein activates SAE1 transcription by directly binding to the E-boxes near the transcription start site of SAE1 [62]. Myc is commonly overexpressed in HCC [63], and may contribute to the high expression of SAE1 in the disease. Besides, microRNAs (miRNAs) are a class of single-stranded, small, and non-protein-coding RNAs that negatively regulate gene expression at the post-transcriptional level [64]. MiR-205 is predicted to target SAE1 via the TargetScan database (http://www.targetscan.org/). MiR-205 has been reported to be downregulated in HCC, and its overexpression suppresses the proliferation of HCC cells [65]. Another study showed that miR-205 could inhibit the migration, invasion, and epithelial–mesenchymal transition (EMT) of HCC cells [66]. Further studies could be directed to the identification of miRNAs in regulating the expression of SAE1 in HCC.

Finally, we established an SAE1-related prognostic model (SRRS) for patients with HCC. SRRS could serve as an independent prognostic factor and be used for predicting the efficiency of TACE treatment. Although TACE is effective for patients with early- and intermediate-stage HCC, more than 40% of patients do not respond to therapy [67,68]. The strong predictability of SRRS in TACE treatment provides clues that patients with HCC having high proliferative capability might not respond to the treatment and that cell cycle inhibitors might provide synergetic effects with TACE treatment for these patients.

## 5 Conclusion

In conclusion, SUMO-2 and SAE1 were upregulated in HCC, and their low levels correlated with the longer survival of patients. SAE1 promoted cell cycle transition and cell proliferation, and its expression was positively regulated by IGF2BP3 in an m6A-dependent way. The established SRRS not only served as an independent prognostic factor but also was helpful in predicting the efficiency of TACE treatment and selecting patients with HCC who might benefit from this treatment.
